# Aberrant cytokeratin 7 expression by hepatocytes can predict the ductopenia grade in primary biliary cholangitis

**DOI:** 10.1186/s12876-022-02538-w

**Published:** 2022-11-02

**Authors:** Hong-Li Liu, An-Yin Yang, Qing-Fang Xiong, Yan-Dan Zhong, Du-Xian Liu, Ping Huang, Xiao-Ning Feng, Yu Zhang, Yong-Feng Yang

**Affiliations:** 1 Southeast University School of Medicine, No87 Dingjiaqiao Road, Gulou District, Nanjing, 210009 China; 2 The Second Hospital of Nanjing, Teaching Hospital of Southeast University, No.1 Zhongfu Road, Gulou District, Nanjing, 210003 China; 3 Department of Liver Diseases, The Second Hospital of Nanjing, Nanjing University of Chinese Medicine, No.1 Zhongfu Road, Gulou District, Nanjing, 210003 China; 4 Department of Pathology, The Second Hospital of Nanjing, Nanjing University of Chinese Medicine, No.1 Zhongfu Road, Gulou District, Nanjing, 210003 China

**Keywords:** Liver cirrhosis, Biliary, Keratin-7, Bile ducts

## Abstract

**Background:**

Aberrant cytokeratin 7 expression by hepatocytes (CK7^+^Hs) is the hallmark characteristic of cholestasis diseases, especially in ductopenia diseases such as primary biliary cholangitis (PBC). This study attempted to evaluate the differences and relationships between the clinical and histological features of aberrant cytokeratin 7 (CK7) expression by hepatocytes in PBC patients.

**Methods:**

The clinicopathological data of patients diagnosed with PBC at the Second Hospital of Nanjing between January 2016 and September 2018 were analysed with SPSS 20.0.

**Results:**

Eighty-nine PBC patients who underwent liver biopsy were enrolled in this study, and 15, 29 and 45 patients had aberrant CK7 expression by hepatocytes (CK7^+^Hs (2 +), CK7^+^Hs (1 +), and CK7^−^Hs, respectively). There were significant differences in TB, DB, ALP, TA, IgM, interface activity, and ductopenia grade between patients with CK7^−^Hs and CK7^+^Hs (2 +) (*P* < 0.05). The ductopenia grade was also significantly different between patients with CK7^+^Hs (2 +) and CK7^+^Hs (1 +) according to sex (*P* < 0.05). Upon merging the data of CK7^+^Hs (2 +) and CK7^+^Hs (1 +) into CK7^+^Hs, we found significant differences in AMA, AMA-M2, anti-gp210, TB, DB, ALP, TA, IgM, fibrosis, and ductopenia grade between CK7^+^Hs and CK7^−^Hs (*P* < 0.05). The odds ratios (ORs) (and 95% confidence intervals (CIs)) of CK7^+^Hs according to anti-gp210, ductopenia grade, and interface activity were 6.413 (95% CI 1.363–30.162), 4.145 (95% CI 1.898–9.052) and 3.247 (95% CI 1.556–6.775), respectively (*P* < 0.05). Spearman's rank correlation according to interface activity and ductopenia grade in patients with CK7^+^Hs (2 + , 1 + , 0) was *r* = 0.359 (*P* = 0.001) and *r* = 0.396 (*P* < 0.001), respectively.

**Conclusion:**

CK7^+^Hs serves as a cholestasis index of PBC and are associated with the ductopenia grade and interface activity. Aberrant cytokeratin 7 expression by hepatocytes can predict the ductopenia grade in primary biliary cholangitis.

## Introduction

Cytokeratins (CKs, keratins) are a series of intermediate filament proteins in epithelial cells and include type I (CK9-20) and type II (CK1-8) proteins according to the conventional classification [1]. They play significant roles in epithelial cells. They not only maintain the structural support of epithelial cells but also regulate cell metabolism (growth, proliferation, migration, apoptosis, immunity and so on). CK7 is an intermediate filament protein with a molecular weight of 54 kDa and is positively expressed in the cytoplasm of cells according to immunohistochemical staining. Furthermore, it is expressed in many epithelial cells of normal tissues, such as breast and lung tissues, and even distinguishes benign from malignant epithelial tumors. Additionally, CK7 is a special marker of bile duct epithelial cells, discerning cancers such as intrahepatic cholangiocarcinoma and hepatocellular carcinoma. Disparities in aberrant CK7 expression by hepatocytes can also sometimes be used to assess the prognosis of cancer patients [2].

In normal liver tissues, it is not difficult to discern hepatocytes from biliary epithelial cells (BECs) with CK. In general, there are no intermediate-positive CK7-expressing cells among these two kinds of cells, even though they are derived from a common fertilized egg. However, CK7-positive hepatocyte-shaped cells, CK7^+^Hs, have been discovered under some liver injury conditions. They are found not only in some paediatric cholestatic diseases [3], such as Alagille syndrome (AGS), extrahepatic biliary atresia (EHBA), progressive familial intrahepatic cholestasis (PFIC), primary sclerosing cholangitis (PSC) and congenital bile acid synthesis errors, but also, in adult patients [4] with hepatitis C virus (HCV) and/or hepatitis B virus (HBV) infection, nonalcoholic steatohepatitis (NASH), alcoholic liver disease (ALD), primary biliary cholangitis (PBC) or overlapping autoimmune hepatitis (AIH). CK7^+^Hs are not confined to the periportal area, which is considered the site of the hepatic progenitor cell (HPC) niche; they also stretch into the centrilobular area [4]. CK7^+^Hs are also found in mice, as in the model of N-diethylnitrosamine-induced hepatocellular lesions [5]. It is speculated that hepatocyte-shaped cells present some characteristics and even perform the functions of BECs. CK7^+^Hs may be relevant to the differentiation of stem cells [4] in liver regeneration or hepatocyte biliary metaplasia during the process of transdifferentiation among BECs and hepatocytes.

CK7^+^Hs are the hallmark of cholestasis diseases [4], especially in ductopenia diseases such as PBC. PBC is an autoimmune liver disease and is associated with middle-aged women, especially those aged approximately 50 years [6]. Autoantibodies are important in the diagnosis of PBC, with AMA (anti-mitochondrial antibody), ‘multiple nuclear dots’ or the ‘rim-like/membranous’ patterns being rare findings. Regardless of the status of anti-mitochondrial antibodies, their positivity indicates the diagnosis of primary biliary cirrhosis. The high specificity of primary biliary cirrhosis makes it a useful diagnostic tool, especially for AMA-negative patients. In addition, anti-gp210 and anti-sp100, as specific antinuclear antibodies (ANA) are also included in the diagnostic criteria. PBC-specific antinuclear antibody ANA expression identified by immunofluorescence, specific anti-sp100/anti-gp210 expression identified Western blotting, or enzyme-linked immunosorbent assay (ELISA) results inform the diagnosis of PBC [7, 8]. Small and mediumsized interlobular bile ducts are usually damaged [9]. The typical lesion is nonsuppurative cholangitis, which indicates that the bile ducts are surrounded by lymphoid follicles and epithelioid granulomas in portal tracts [10]. Bile duct injury progresses, and the small intrahepatic bile duct is lost. However, larger bile ducts may present only inflammation and epithelial damage [11]. The manifestation of ductopenia is that the small artery is not accompanied by the bile duct in the portal tract, and the contacts between hepatocytes and bile ducts are interrupted by the loss of bile ducts [11]. As a result, histological changes take place. CK7 is used to assess the stage and mark the bile ducts [9, 12]. This is especially true for those infiltrated with a large number of lymphocytes, as bile ducts are rarely seen in the portal tracts. However, sometimes CK7^+^Hs are discovered, especially during ductopenia. However, it is unclear whether there are other differences and relationships between the clinical and pathological features and degree of CK7^+^Hs in PBC patients.

In this article, we report the clinical and histological factors that are related to the different degrees of CK7-positive hepatocytes (CK7^+^Hs) in PBC, and we discuss other features of PBC.

## Materials and methods

### Patient selection

We retrospectively selected patients who were diagnosed with PBC via liver biopsy at the Second Hospital of Nanjing between January 2016 and September 2018. In this retrospective study, the data were anonymous, and the requirement for informed consent was waived by the Medical Ethics Committee of the Second Hospital of Nanjing,. (No. 2020-ly-ky030). This study was approved by the examination of the Second Hospital of Nanjing,. (No. 2019-ly-kt073). The study protocol conformed to the ethical guidelines of the Declaration of Helsinki.

### Inclusion and exclusion criteria

#### Inclusion criteria

1. Patients who underwent liver biopsy and staining with haematoxylin and eosin (HE) stains and immunohistochemistry markers (CK7 and CD38).
2. Patients who met the criteria of the *Consensus on the diagnosis and management of primary biliary cirrhosis (cholangitis) of China in 2015* [13]. At least two of the three items below were satisfied:
  - Liver function tests showed a pattern of cholestasis, such as an increase in alkaline phosphatase (ALP).
  - The expression of anti-mitochondrial antibody (AMA) and/or AMA-M2 was positive.
  - Pathological lesions were consistent with typical changes in PBC.

#### Exclusion criteria

1. The total number of portal tracts in a histopathological section was less than five under a microscope.
2. The expression of CK7 was a false negative, and the bile duct was observed with HE but not CK7 staining

### Collection of clinical data

Age, sex, diagnosis, liver function test, autoimmune antibody, and immunoglobulin data were collected.

### Reading of pathological sections

**Fig. 1:**
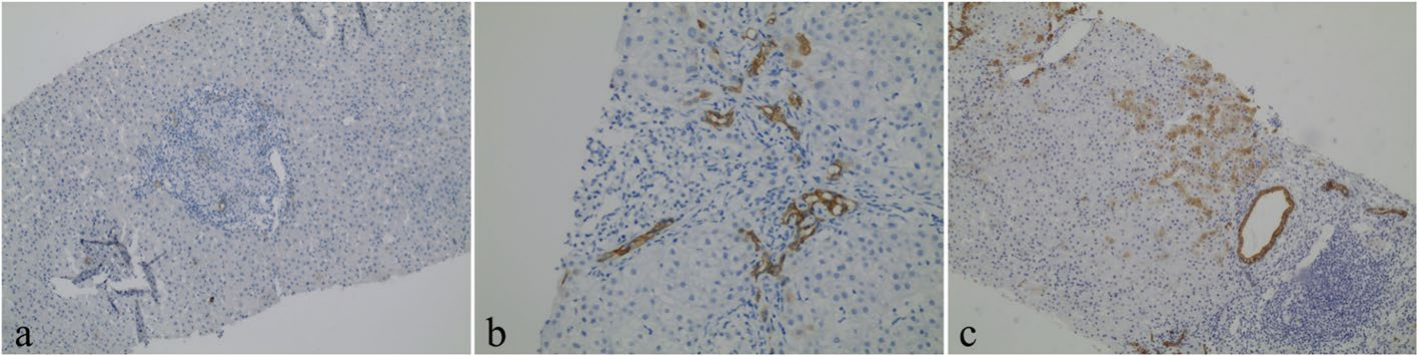
Aberrant CK7 expression by hepatocytes according to the immunohistochemical staining scores of PBC patients. (expression of CK7 positive hepatocytes in lobules). **a** 0 = absence (original magnification × 100, immunohistochemical staining of CK7); **b** 1 = isolated hepatocytes were positive (original magnification × 200, immunohistochemical staining of CK7); and **c** 2 = cluster/diffused hepatocytes were positive (original magnification × 100, immunohistochemical staining of CK7)

1. Two doctors made a joint assessment of the histological lesions under a microscope together without accessing the clinical information.
2. The number of complete or nearly complete (accounting for at least 80% of the area) portal tracts in each specimen was recorded.
3. Evaluated characteristics based on semiquantitative scales: Table 1 and Fig. 1.
  - HE staining: fibrosis (1, 2, 3, and 4); interface activity (0, 1, 2, and 3); and ductopenia grade (1, 2, 3, and 4).
  - CD38 staining: plasma cell infiltration (0, 1, 2, and 3).
  - CK7 staining: ductular reaction (0, 1, and 2); CK7-positive hepatocytes (0, 1, and 2).

**Table 1:** Histological assessment score in PBC patients

Feature	Score
Fibrosis	
Fibres were not seen or confined to the portal tracts	1
Fibres extended from the portal tracts, but there was no bridging	2
Fibres were bridging, but lobules were still clear	3
Liver cirrhosis and pseudo lobules were noted	4
Interface activity	
No: the boundary plate was complete	0
Mild: the inflammatory damage involved less than 30% of the interface	1
Moderate: -the inflammatory damage involved 30%-50% of the interface	2
Severe: the inflammatory lesions involved more than 50% of the interface	3
Ductopenia grade	
The proportion of missing bile ducts in the theoretical number of bile ducts in the portal area was less than 25%	1
The proportion of missing bile ducts in the number of theoretical bile ducts in the portal area was greater than or equal to 25% but less than 50%	2
The proportion of missing bile ducts in the number of theoretical bile ducts in the portal area was greater than or equal to 50% but less than 75%	3
The proportion of missing bile ducts in the number of theoretical bile ducts in the portal area was greater than or equal to 75%	4
Plasma cells (CD38 staining)	
No: absence	0
Few: isolated cells were positive	1
Middle: clustered cells were positive	2
Many: diffuse cells were positive	3
Ductular reaction (DR, CK7 staining)	
No: absence	0
Few: less than 50% of the portal tracts	1
Many: more than 50% of the portal tracts	2
Aberrant CK7 expression by hepatocytes	
Absence	0
Isolated hepatocytes were positive	1
Clustered/diffuse hepatocytes were positive	2

### Statistical analyses

Statistical analyses were performed with SPSS 20.0 and GraphPad Prism 8. Variables with skewed distributions are expressed as the range and median, while variables with normal distributions are expressed as the mean ± standard deviation. The clinical data and some pathological features were analysed by parametric tests such as the t test and analysis of variance (ANOVA) and nonparametric tests such as the chi-square test, Kruskal‒Wallis test, and Spearman rank correlation (according to data type). The data were also analysed by Bonferroni, Student‒Newman‒Keuls, Kruskal–Wallis one‒way ANOVA and Mann‒Whitney U tests to compare differences among more than two groups. Then, binary logistic regression analysis was performed to identify the risk factors for CK7 + Hs. A *P* value < 0.05 was considered statistically significant.

## Results

### Selection of patients

Eighty-nine PBC patients (16 males and 73 females) who underwent liver biopsy were enrolled in this study. Forty patients had simple PBC, and forty-nine patients had overlapping AIH. Their ages ranged from 27 to 74 years.

### Clinical and pathological data

Table 2 shows the clinical and pathological information of hepatocytes with different degrees of aberrant CK7 expression. The numbers of patients with CK7^+^Hs (2 +), CK7^+^Hs (1 +), and CK7^−^Hs were 15, 29, and 45, respectively. The results of autoimmune antibody tests could be dated back in 85 patients, and 50 and 51 individuals had positive expression of AMA and AMA-M2, respectively. Twenty-one patients were positive for the anti-gp210 antibody(immunoblotting), and 15 patients were positive for the anti-sp100 antibody (immunoblotting).There were significant differences in TB, DB, ALP, TA, IgM, interface activity, and ductopenia grade between CK7^−^Hs and CK7^+^Hs (2 +) (*P* < 0.05). The ductopenia grade was also significantly different between patients with CK7^+^Hs (2 +) and CK7^+^Hs (1 +) according to sex (*P* < 0.05). Upon merging the data on CK7^+^Hs (2 +) and CK7^+^Hs (1 +) (into CK7^+^Hs), there were significant differences in AMA, AMA-M2, anti-gp210, TB, DB, ALP, TA, IgM, fibrosis, and ductopenia grade (*P* < 0.05).

**Table 2:** Clinicopathological comparisons of patients with cytokeratin 7-expressing hepatocytes

Index	Patients with CK7^+^Hs(*n* = 44)		Patients with CK7^−^Hs (*n* = 45)	P_1_	P_2_
	Patients with CK7^+^Hs (2 +) (*n* = 15)	Patients with CK7^+^Hs (1 +) (*n* = 29)			
Age mean ± SD	50.79 ± 5.75	50.67 ± 9.45	53.24 ± 10.47	0.521^‡^	0.258^†^
Sex (female⁄ male)	15/0^*^	19/10^*^	39/6	0.010^††^	0.249 ^¶^
Diagnosis PBC/OS	4/11	12/17	24/21	0.178^††^	0.108^¶^
AMA ( ±)	12/3	19/10	19/22 (*n* = 41)	0.051^††^	0. 024^¶^
AMA-M2 ( ±)	12/3	19/10	20/21 (*n* = 41)	0.081^††^	0.042^¶^
Anti-gp210 ( ±)	6/9	9/20	6/35 (*n* = 41)	0.093^††^	0.038 ^¶^
Anti-sp100 ( ±)	2/13	8/21	5/36 (*n* = 41)	0.223^††^	0.203^¶^
ANA ( ±)	8/7	13/16	24/17 (*n* = 41)	0.527^††^	0.318^¶^
TB range (median) umol/L	9.9–277.1 (24.0)*	7.3–337.6 (18.1) (*n* = 28)	8.1–341.1 (13.2)^*^ (*n* = 41)	0.005 ^§^	0.005 ^§^
DB range (median) umol/L	2.8–196.7 (13.0)* (*n* = 14)	2.2–249.8 (7.7) (*n* = 28)	2.1–254.6 (4.4)^*^ (*n* = 41)	0.004 ^§^	0.004 ^§^
Alb mean ± SD g/L	39.40 ± 6.17 (*n* = 14)	38.08 ± 5.77 (*n* = 28)	40.51 ± 4.71 (*n* = 40)	0.246^‡^	0.112^†^
ALT range (median) IU/L	20.0–846.3 (58.7)	13–664.4 (64.9) (*n* = 28)	15.7–1062.3 (61.1) (*n* = 41)	0.772^§^	0.421 ^§^
AST range (median) IU/L	38.4–646.1 (92.3)	20.8–473.1 (68.05) (*n* = 28)	17.4–1392 (58.7) (*n* = 41)	0.152 ^§^	0.134 ^§^
ALP range (median) IU/L	117.6–766.3 (347.5)^*^ (*n* = 14)	77.2–750.6 (205) (*n* = 27)	73.7–684.1 (168.25)* (*n* = 40)	0.002 ^§^	0.005 ^§^
γ-GT range (median) U/L	27.9–1840.8 (323.3) (*n* = 14)	22.4–638.8 (214.5) (*n* = 27)	22.5–799.1 (188.85) (*n* = 40)	0.084 ^§^	0.054 ^§^
TA range (median) umol/L	2.4–369.9 (45.4)* (*n* = 14)	3.7–389.1 (26.5) (*n* = 28)	1.1–244.1 (15.9)* (*n* = 40)	0.006 ^§^	0.005 ^§^
IgA mean ± SD g/L	2.84 ± 1.39	3.07 ± 1.25 (*n* = 25)	2.80 ± 0.93 (*n* = 40)	0.856^‡^	0.641^†^
IgG mean ± SD g/L	16.54 ± 5.13	15.48 ± 4.62 (*n* = 25)	16.06 ± 4.90 (*n* = 40)	0.631^‡^	0.961^†^
IgM mean ± SD g/L	3.47 ± 1.83^*^	3.13 ± 1.56 (*n* = 25)	2.31 ± 1.42^*^ (*n* = 40)	0.026^‡^	0.011^†^
Fibrosis (1/2/3/4)	2/6/4/3	5/11/9/4	17/14/13/1	0.056 ^§^	0.018 ^§^
IA (0/1/2/3)	1/3/5/6^*^	1/11/12/5	11/17/14/3^*^	0.003 ^§^	0.002 ^§^
D grade (1/2/3/4)	3/3/7/2^*#^	9/8/11/1^*^	26/13/5/1^#^	0.001 ^§^	< 0.001^§^
DR (0/1/2)	3/8/4	4/18/7	19/13/13	0.354 ^§^	0.152 ^§^
Plasma cells (0/1/2/3)	0/8/6/1	0/13/12/4	4/24/12/5	0.255^§^	0.125 ^§^

*CK7*^+^*Hs (2* +*)* Cytokeratin 7-positive hepatocytes grade 2, *CK7*^+^*Hs (1* +*)* Cytokeratin 7-positive hepatocytes grade 1, *CK7*^+^*Hs* Cytokeratin 7-positive hepatocytes, *CK7*^*−*^*Hs* Cytokeratin 7-negative hepatocytes, *PBC* Primary biliary cholangitis, *OS* Overlap syndrome, *AMA* Anti-mitochondrial antibody, *AMA-M2* Anti-mitochondrial antibody-M2, *anti-gp210* Anti-glycoprotein 210(immunoblotting), *anti-sp100* Anti-soluble protein 100(immunoblotting), *TB* Total bilirubin, *DB* Direct bilirubin, *Alb* Albumin, *ALT* Alanine aminotransferase, *AST* Aspartate aminotransferase, *ALP* Alkaline phosphatase, *γ-GT* γ-glutamyl transferase, *TA* Total bile acid, *IgA* Immunoglobulin A, *IgG* Immunoglobulin G, *IgM* Immunoglobulin M, *IA* Interface activity, *D grade* Ductopenia grade, *DR* Ductular reaction

P_1_: *P* value in CK7 + (2, 1, 0), P_2_: *P* value in CK7 (+ , -)

^†^ unpaired t test

^‡^ one-way-ANOVA

^§^ Kruskal‒Wallis test

^¶^ Mann‒Whitney U test

^††^ chi-square test

^#*^ indicates a significant difference between two groups

### Binary logistic regression analysis of the clinical and pathological data

The variables that showed significant or nearly significant differences when comparing the two groups based on the positive or negative expression of CK7 by hepatocytes (Table 2) were analysed via binary logistic regression and included the following: AMA, AMA-M2, anti-gp210, TB, DB, ALP, gamma-glutamyl transferase (GGT), TA, fibrosis, interface activity and ductopenia grade. The Froward Wald examination was used in the binary logistic regression analysis, and the final data included in the equation are shown in Table 3.

**Table 3:** Binary logistic regression analysis of risk factors associated with cytokeratin 7-positive hepatocytes among the clinicopathological features

	B	SE	Wald	df	Sig	Exp (B)	95% CI for EXP (B)	
							Lower	Upper
Anti-gp210	1.858	0.790	5.534	1	0.019	6.413	1.363	30.162
Ductopenia grade	1.422	0.399	12.723	1	0.000	4.145	1.898	9.052
Interface activity	1.178	0.375	9.843	1	0.002	3.247	1.556	6.775
Constant	-4.947	1.205	16.842	1	0.000	0.007		

The final variables included in the equation were anti-gp210, ductopenia grade, and interface activity, as they were risk factors for CK7^+^Hs. When other factors were fixed, the odds ratios (ORs) (and 95% confidence intervals (CIs)) of CK7^+^Hs according to anti-gp210, ductopenia grade, and interface activity were 6.413 (95% CI 1.363–30.162), 4.145 (95% CI 1.898–9.052) and 3.247 (95% CI 1.556–6.775), respectively (*P* < 0.05). AMA, AMA-M2, TB, DB, ALP, GGT, TA and fibrosis were not risk factors for CK7^+^Hs

### Distribution of interface activity and ductopenia grade in patients with CK7^+^Hs

**Fig. 2:**
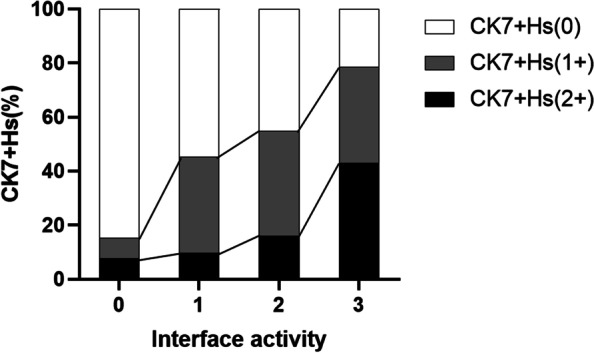
Distribution of interface activity in patients with CK7^+^Hs. (interface activity 0, 1, 2, and 3: no, mild, modest, and severe; CK7^+^Hs: cytokeratin 7-positive hepatocytes)

**Fig. 3:**
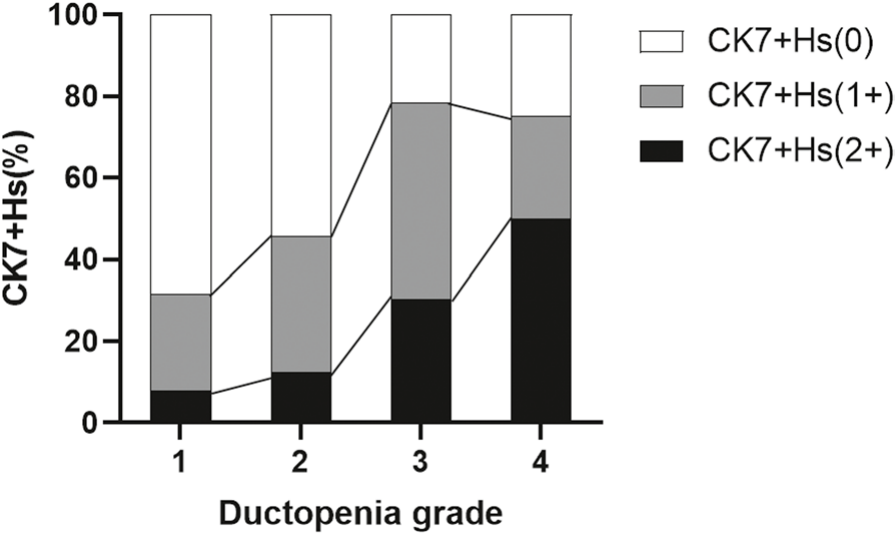
Distribution of the ductopenia grade in patients with CK7^+^Hs. (ductopenia ratio 0, 1, 2, and 3: 0–25%, 25%-50%, 50%-75%, and 75%-100%; CK7.^+^Hs: cytokeratin 7-positive hepatocytes)

Spearman’s rank correlations according to interface activity and the ductopenia grade in the CK7^+^Hs (2 + , 1 + , 0) group were *r* = 0.359 (*P* = 0.001) and *r* = 0.396 (*P* < 0.001), respectively. The percentage of interface activity and ductopenia grade according to the expression of CK7^+^Hs are shown as stacked column charts in Figs. 2 and 3 (*P* < 0.05). There was a gradual increase in the expression of CK7^+^Hs by hepatocytes with increasing interface activity, and the percentage of CK7^+^Hs (2 +) also increased, as shown in Fig. 2. Similarly, there was a gradual increase in the expression of CK7^+^Hs by hepatocytes with increasing ductopenia grade. However, there was a slight decrease in grade 4 ductopenia, while the percentage of CK7^+^Hs (2 +) increased continuously.

## Discussion

PBC is an intrahepatic cholestasis disease that damages < 100 μm interlobular bile ducts of the biliary tree. The bile ducts are composed of cuboidal cells with a basement membrane and are easily identified by CK7 immunohistochemical staining. There are some histological staging systems for PBC, such as Scheuer’s and Ludwig’s systems, thatdescribe the histology by inflammation and fibrosis. However, it is not reasonable to ignore bile duct injury according to the pattern of the system, which is not parallel to inflammation or fibrosis. Therefore, Nakanuma [14] established a scoring system to evaluate the histological features of PBC, including fibrosis, bile duct loss and chronic cholestasis (deposition of orcein-positive granules). Because the pathological changes in PBC are extremely uneven, some patients possess only one typical portal tract characteristic, such as a florid bile duct and lymphocyte follicles. Other researchers have found that the CK7 score in liver tissues can discriminate the stage of PBC better than the CK19 score.Additionally some hepatocytes are stained by CK7 rather than CK19 [15], although both kinds of CK have the ability to mark the bile ducts.

In this study, we explored the clinical and pathological differences associated with different degrees of CK7^+^Hs in 89 liver specimens from patients diagnosed with PBC, 49 of whom had overlapping AIH. It is difficult to diagnose overlap syndrome, because the IAIHG cumulative score used to identify AIH has not been validated in this particular setting [16]. However, in this study, overlap syndrome patients were diagnosed by Paris criteria [16, 17]. This study demonstrated that anti-gp210, interface activity, and ductopenia grade are risk factors for CK7^+^Hs in PBC. Interface activity and ductopenia grade were positively correlated with CK7^+^Hs. Moreover, we found that TB, DB, ALP, and TA were different between CK7^−^Hs and CK7^+^Hs (2 +), showing that the cluster or diffuse distributions of CK7^+^Hs indicate cholestasis of the liver. Previous studies have shown that ductopenia is related to CK7^+^Hs, but in this study, we showed that anti-gp210 and interface activity are risk factors for CK7^+^Hs.

There were differences in AMA, AMA-M2, and anti-gp210 between the CK7^+^Hs and CK7^−^Hs groups in this article. However, some patients may not exhibit typical staining patterns for AMA or AMA-M2, and they need liver biopsy to diagnose PBC.Thus, the difference may be related to this reason. Anti-gp210 is an index that can be used to help diagnose AMA-negative PBC [18] and evaluate the severity or prognosis of PBC [19]. It is also a risk factor for CK7^+^Hs. There were significant differences in patients with CK7^+^Hs (2 +) and CK7^+^Hs (1 +) according to sex; however, the number of men with CK7^+^Hs (2 +) was zero. Therefore, the sample size needs to be increased to confirm this finding in the future. In addition, fibrosis was significantly different between the CK7^−^Hs and CK7^+^Hs groups (*P* = 0.018) but not between the CK7^+^Hs (2 +), CK7^+^Hs (1 +), and CK7^−^Hs groups (*P* = 0.056) (close to 0.05). Differences among the three groups may also be observed if we expand the subject. As the disease progresses, the bile duct is gradually damaged, and fibres extend from one portal tract to another portal tract or central lobule.Eventually bridging fibres and cirrhotic nodules are formed. Furthermore, there were differences in interface activity, in which the amount of CK7^+^Hs increased with increasing interface activity. Interface activity may tend to be a feature of overlap syndrome (OS) because PBC possesses only mild lymphocyte interface activity. During this process, inflammatory cells can spill over from the portal tracts into the adjacent parenchyma or biliary interface changes at the plate between portal tracts and parenchyma take place. Therefore, damage to the interface plate is limited to PBC, and OS is considered when there is a prominent lymphocytic interface or lobular inflammation.

CK7^+^Hs are hepatocyte-shaped cells., Not only is the nuclear/cytoplasm ratio lower than that of BECs, but staining for CK7^+^Hs and ductular reactions are also weaker. The main feature of CK7^+^Hs, especially in ductopenia patients, is considered to be cholestasis. Matsukuma [4] found that GGT was associated with CK7^+^Hs; however, we discovered that TB, DB, ALP, and TA were different between CK7^−^Hs and CK7^+^Hs (2 +). In addition, IgM, another typical index of PBC, also showed differences between CK7^−^Hs and CK7^+^Hs (2 +). The increase in ALP should be connected with AMA or typical liver lesions when diagnosing PBC, and the degree of ALP elevation may be associated with different outcomes that are closely related to the severity of ductopenia and inflammation in cirrhosis. IgM is an antibody secreted by plasma cells; however, we did not discover differences in plasma cells that were stained with CD38 in liver tissues in our experiments. This finding may be because the subjects included AIH patients, in whom plasma cells may also secrete IgG in tissues.Thus, the results would be inconsistent. The differences between CK7^−^Hs and CK7^+^Hs (2 +) indicate that the cluster or diffuse distributions of CK7^+^Hs may be related to the cholestasis index. In addition, the cholestasis index may be associated with ductopenia and reduce the connections between the biliary tree and hepatocytes. In normal tissues, hepatocytes possess three specialized membrane domains.Tthe basolateral or sinusoidal domainis the vascular pole that faces the sinusoids.The lateral membrane of the cell is made up of two domains: the canalicular domain and the lateral domain. The canalicular domain, along with the canicular domain of the adjoining hepatocyte, makes up the bile canaliculus and is also called the biliary or apical pole of the hepatocyte. The bile secreted by hepatocytes flows from the bile ductile to bile canaliculi; finally, it gathers to in the IBID. When the bile duct is lost, the link between hepatocytes and IBID is interrupted. This may be related to cholestasis. The body will change to alleviate the lesions, and CK7^+^Hs may be observed.

Currently, the mechanisms of CK7^+^Hs are not clear. Some studies have illustrated that aberrant CK7 expression by hepatocytes originates from the canals of Hering, where HPCs differentiate into hepatocytes and cholangiocytes when the periportal areas between portal tracts and parenchyma are damaged [20]. HPCs are similar to oval cells in rodents, and the ductular reaction (DR) is also believed to originate from HPCs. DR is usually the earliest change to occur when the bile duct without cavity structures is injured. It provides a bypass and promotes the movement of bile fluid from the small-diameter bile duct to the predominate bile duct during damage to or loss of BECs. CK7^+^Hs may be another product of DR and possess a similar morphology to hepatocytes during regeneration of the liver. However, CK7 staining is weaker than that of BECs. Although HPCs are usually located around periportal areas, CK7^+^Hs in centrilobular hepatocytes can sometimes be found.This may be another niche for migrating HPCs [21].

Other studies have suggested that CK7^+^Hs is related to metaplasia, which is transdifferentiated by mature hepatocytes [22] by signalling pathways, such as the Notch and TGF-β pathways. Some researchers [23] have found transdifferentiation from hepatocytes to cholangiocytes during severe biliary damage and established a chimeric liver model. In this model, dipeptidyl peptidase (DPP)IV- rats were transplanted with hepatocytes from DPP IV  + rats and underwent bile duct ligation (BDL) following pretreatment with 4,4’-methylenedianiline (DAPM). Approximately 47.5% DPP IV  + cholangiocytes were observed in the chimeric rat liver. Other researchers also found negative correlations between changes in hepatocytes and BECs.The expression of CK7( +) was increased in BDL rats [24, 25] which showed that the positive expression of Hepar in hepatocytes was decreased. In addition, some researchers consider the process of CK7^+^Hs to occur inthe middle process during of the transdifferentiation from mature hepatocytes to cholangiocytes. Sato et al. [26] introduced the mechanism of DR. They elucidated that mature hepatocytes could change to hepatocytes and that DR was also derived from the transdifferentiation of hepatocytes.

There are some limitations to this study. The total number of PBC patients who had to undergo liver biopsy for diagnosis and treatment was too small to use ordinal regression to investigate the relationships among the degree of CK7^+^Hs in PBC. Some patients can be diagnosed without liver biopsy for classic PBC. However, some of them need a biopsy to assist in diagnosis and treatment. For example, patients with staging and grading of PBC, such as antibody-negative PBC, and patients with poor Ursodeoxycholic Acid (UDCA) treatment, overlapping autoimmune hepatitis, and differentiation from other liver diseases. Moreover, the mechanism of CK7^+^Hs needs further study. The sample size needs to be increased to confirm the data and explore the mechanisms of CK7^+^Hs in the future.

## Conclusion

In this study, we conclude that the expression of CK7 + Hs is a histologic feature of PBC, in which cluster or diffuse diffusion is a sign of cholestasis. Additionally CK7 + Hs are positively correlated with the ductopenia grade and interface activity in PBC patients. The risk factors for aberrant CK7 expression by hepatocytes are anti-gp210, ductopenia grade and interface activity. However, the mechanisms related to these hepatocytes needs further study, but it may be related to biliary metaplasia or the differentiation of HPCs. Aberrant cytokeratin 7 expression by hepatocytes can predict the ductopenia grade in primary biliary cholangitis.

## Data Availability

The datasets used and analysed during the current study are available from the corresponding author on request.

## Abbreviations

CK7^+^Hs: Aberrant cytokeratin 7 with hepatocytes
PBC: Primary biliary cholangitis
CKs: Cytokeratins
BECs: Biliary epithelial cells
AGS: Alagille syndrome
EHBA: Extrahepatic biliary atresia
PFIC: Progressive familial intrahepatic cholestasis
PSC: Primary sclerosing cholangitis
HCV: Hepatitis C virus
HBV: Hepatitis B virus
NASH: Nonalcoholic steatohepatitis
ALD: Alcoholic liver disease
PBC: Primary biliary cholangitis
AIH: Autoimmune hepatitis
HPC: Hepatic progenitor cell
HE: Haematoxylin and eosin
ALP: Alkaline phosphatase
AMA: Anti-mitochondrial antibody
OS: Overlap syndrome
AMA: Anti-mitochondrial antibody
AMA-M2: Anti-mitochondrial antibody-M2
anti-gp210: Anti-glycoprotein 210
anti-sp100: Anti-soluble protein 100
TB: Total bilirubin
DB: Direct bilirubin
Alb: Albumin
ALT: Alanine aminotransferase
AST: Aspartate aminotransferase
ALP: Alkaline phosphatase
γ-GT/GGT: γ-Glutamyl transferase
TA: Total bile acid
IgA: Immunoglobulin A
IgG: Immunoglobulin G
IgM: Immunoglobulin M
IA: Interface activity;
D grade: Ductopenia grade
DR: Ductular reaction
ORs: Odds ratios
CIs: Confidence intervals
UDCA: Ursodeoxycholic Acid
ANA: Anti nuclear antibody
IAIHG: International Autoimmune Hepatitis Group
